# Chromosome Visualization Tool: A Whole Genome Viewer

**DOI:** 10.1155/2011/373875

**Published:** 2011-12-19

**Authors:** Ethalinda K. S. Cannon, Steven B. Cannon

**Affiliations:** ^1^Department of Genetics, Development, and Cell Biology, Iowa State University, Ames, IA 50011, USA; ^2^United States Department of Agriculture-Agricultural Research Service, Corn Insects and Crop Genetics Research Unit, Ames, IA 50011, USA

## Abstract

CViT (chromosome visualization tool) is a Perl utility for quickly generating images of features on a whole genome at once. It reads GFF3-formated data representing chromosomes (linkage groups or pseudomolecules) and sets of features on those chromosomes. It can display features on any chromosomal unit system, including genetic (centimorgan), cytological (centiMcClintock), and DNA unit (base-pair) coordinates. CViT has been used to track sequencing progress (status of genome sequencing, location and number of gaps), to visualize BLAST hits on a whole genome view, to associate maps with one another, to locate regions of repeat densities to display syntenic regions, and to visualize centromeres and knobs on chromosomes.

## 1. Introduction

Visualizing features on a whole genome (all chromosomes together) can be informative for many reasons: for identifying genome-wide patterns such as gene or repeat densities, for viewing internal duplications or synteny, for assessing clustering of genes or repeats or other features, for comparing chromosomal structures such as centromeres and pericentromeric regions, or for looking for associations between different types of genomic features. Several very capable genome browsers enable visualization of single chromosomes or regions, but few visualization tools have been developed for whole-genome-at-a-time views. We present CViT (chromosome visualization tool), for viewing a wide range of genomic features on an arbitrary set of linear regions—typically, all of the chromosomes or linkage groups for a genome.

CViT is a set of Perl scripts that generate a PNG (portable network graphics) image of features on chromosomes. It can be executed as a standalone Unix command line utility or wrapped in a web page for either static display or as part of an interactive online tool. The characteristics of the output images are highly configurable. A package containing the CViT code itself along with documentation, examples, supporting scripts, and sample web implementations can be freely downloaded from SourceForge at http://sourceforge.net/projects/cvit/.

CViT was initially developed to support the *Medicago truncatula* sequencing project [1] where it was used to display the assembled bacterial artificial chromosomes (BACs) and the status of the sequencing for each BAC. CViT was also wrapped in web pages to create interactive tools: to display BLAST [2] hits on the whole genome (see http://www.medicagohapmap.org/advanced_search_page.php?seq) and to search where BACs of interest are anchored on the pseudomolecules. For other projects, it has also been used to display genetic maps and contig assemblies from related species [3] and to correlate genetic and cytogenetic maps (http://planthub.gdcb.iastate.edu/lawrencelab/Morgan2McClintock/Version3.0/) [4]. It has been integrated into model organism databases at the Medicago genome sequencing and HapMap projects (http://medicago.org/ and http://medicagohapmap.org/) [5], at MaizeGDB (http://maizegdb.org/) [6], at the PrOject Portal for corn (POPcorn: http://popcorn.maizegdb.org/) [7], and at the legume-family clade database, the Legume Information System (LIS: http://comparative-legumes.org/) [8]. It has also been used to generate analyses and images for publication on genome structure and evolution [9–11].

**Figure 1:**
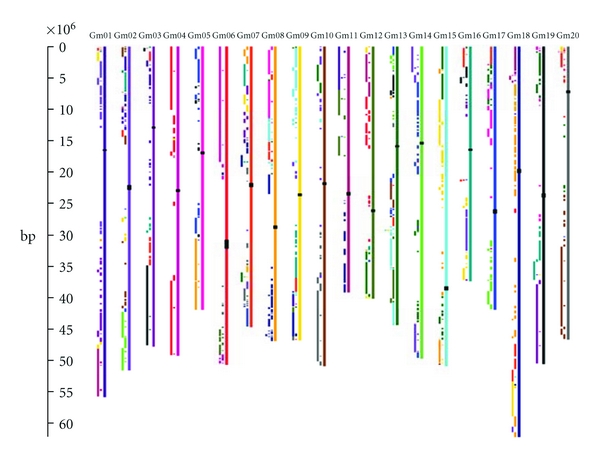
Duplicated segments within the soybean (*Glycine max*) genome. Colored blocks to the left of each chromosome show regions of correspondence with chromosomes of the same color. For example, the light blue blocks at the top of Gm09 correspond with regions on the light blue Gm15, and vice versa. These correspondences are remnants after the *Glycine* genome duplication. Locations of centromeric repeats are shown as black rectangles over the chromosomes. Regions lacking internal correspondences (generally near chromosome centers) mark the approximate locations of the gene-poor pericentromeres. This figure is modified from the Legume Information System, where sequence-based searches can be made against the *Glycine max*, *Medicago truncatula*, and *Lotus japonicus* genomes, with CViT images displaying the sequence homologies and the synteny relationships among these genomes.

Several other whole genome viewers exist, but each has a different set of capabilities than CViT. One example of a whole genome viewer is Flash GViewer (http://gmod.org/wiki/Flash_GViewer), which was developed by the GMOD project (including the GBrowse browser). GViewer is entirely web based (being implemented in Flash), whereas CViT can be used either as a standalone command line utility or embedded in a web page. GViewer also uses an XML format for its data, while CViT uses GFF-formated data to make it compatible with GBrowse and other browsers. Being implemented in Flash, GViewer slows down when many (100 s) of features are displayed. CViT can usually display several thousand features, as the features are placed on a raster image and then handed off to the web client. GViewer is an interactive tool, while CViT requires additional web programming to be made interactive in a web page; on the other hand, GViewer does not produce images for publication, which is a key use for CViT. CViT also shares some of the capabilities of another whole genome viewer, the CIRCOS genome data visualizer [12], with the primary exception being the linear chromosome layout in CViT and circular layout in CIRCOS. The circular layout in CIRCOS facilitates display of relationships between chromosomes, through arcs that travel within the circle that is circumscribed by the ring of chromosomes. CViT is also capable of displaying within- or between-genome synteny relationships, as shown in Figure 1, but may have a greater strength in displaying other kinds of features, where the linear chromosome layout allows separation of features across whatever linear scale has been selected. In this respect, CViT bears some resemblance to genome browsers such as GBrowse [13], the Ensembl Genome Browser [14], IGB (the Integrated Genome Browser), Artemis [15], and the UCSC genome browser [16]—although those browsers are designed primarily for close, interactive examination of single chromosomes at various scales rather than large scale patterns. CViT may therefore often be useful for providing a genome-wide overview, with the task of close, interactive examination of single chromosomal regions left to one of the genome browsers above. This has been done, for example, in the implementation at LIS, where BLAST hits displayed in CViT link to 100 kb GBrowse windows around the hit in the browser for the corresponding genome.

The GFF3 data format (http://www.sequenceontology.org/gff3.shtml), referred to from here on as just “GFF,” was selected because of its ease of use and effective representation of genetic and genomic information and also because it enables sharing data with GBrowse [13] and other GFF-capable browsers. As much as possible, we used GBrowse data and configuration conventions to enable display of the same data in both CViT and GBrowse.

Add-on scripts are provided in the download package to aid in preparation of GFF files—for example, for conversion from BLAST output to GFF. In addition, some web implementations are provided which can be used as is or modified to fit specific needs or to serve as examples of how CViT could be used as part of a larger online resource.

## 2. Implementation

CViT consists of a package of Perl scripts along with a set of add-on scripts and some basic web implementations written in PHP. It requires libgd (http://bitbucket.org/pierrejoye/gd-libgd) and the GD Perl packages GD and GD::Arrow (http://search.cpan.org/dist/GD/). It has been tested on several Linux, Unix, and Apple OS X platforms and is expected to be operational on any Unix variant that can run Perl and libgd.

The input data, in GFF format, must at a minimum contain information about at least one chromosome. All features are related to the chromosome(s) by the value in the first (seqid) column. The coordinates of each feature must lie within the start and end coordinates of its chromosome. Features can be named with the “name” attribute in the last (attribute) column, with the name optionally displayed on the image, and grouped together with the “class” attribute, with each class of features displayed in a different color.

The term “chromosome” here refers to the backbone used to display features. It could in fact be a linkage group, pseudomolecule, BAC, contig, gene, or any stretch of DNA or genetic sequence upon which features can be placed. Similarly, the coordinate system can be based on any unit of measure, such as base pairs, centimorgans, centiMcClintocks [4], or microns.

A “feature” can be just about anything that can be associated with “chromosome” coordinates—for example, centromeres, markers, BACs, BLAST hits, repetitive elements, or gene loci. Feature densities (such as for genes or repetitive elements) can also be displayed using the histogram glyph.

CViT output includes three files: a PNG image displaying the chromosomes and features, a legend image describing the feature glyphs, and a file of feature names and coordinates where they are located on the image. The coordinate file can be used to create interactive web pages—for example, to create an HTML image map to enable clicking on features to get more information about them or to link out to other online resources.

Manipulation of the output image is enabled by a simple but extensive configuration file. This file enables control of almost all aspects of the output image without touching the code, including selecting fonts for labels. Two freely available True Type fonts are included in the download package and any True Type Font can be added. Colors, transparency, sizes of the glyph, location of glyphs relative to the chromosome, location of their text labels, and the appearance of the chromosomes themselves and their spacing are all under configuration control.

Add-on scripts include blastp_to_gff.pl, which generates a GFF file for CViT if provided a GFF file of peptides and either a tabular BLAST output file or a two-column hash of query IDs and peptide IDs, and clusterHSP.pl, which collapses adjacent BLAST HSPs that occur within a sliding window (often appropriate for a peptide or cDNA query against pseudomolecule sequences). The web implementations provided with the package include a simple “CViT-BLAST” implementation which can be used as is or modified to fit specific needs. There is also a web utility named “CViT-web” which provides a web interface for generating CViT images. For some users this may be easier than modifying the configuration file. 

## 3. Biological Examples

Examples of two online instances of CViT are the soybean, *Medicago truncatula*, and *Lotus japonicus* genomes at LIS (http://comparative-legumes.org/) and the maize genome at MaizeGDB (http://maizegdb.org/).

The display of the soybean genome (Figure 1) illustrates the use of CViT for showing internal synteny (chromosomal correspondences) from a whole genome duplication that is estimated to have occurred in the Glycine genus between ~5 and 13 Mya [19, 20]. The implementation at LIS also shows correspondences between the three sequenced genomes (in addition to the correspondences within the duplicated soybean genome). The LIS implementation also allows a sequence search with a multi-FASTA file. Sequence matches (BLAST hits) are color coded and link out to the genome browser for the target genome, with browser views centered around each hit.

**Figure 2:**
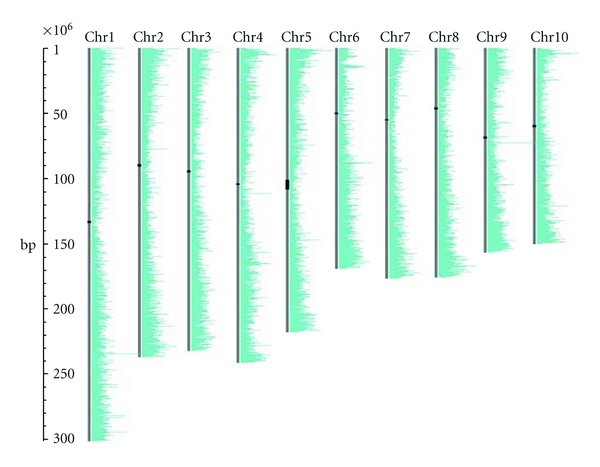
Gene density on the 10 chromosomes of *Zea mays*. Gene density is shown on the *Zea mays* inbred line B73 RefGen_v2 genome assembly [17]. Probable locations of the centromeres are displayed as black bars positioned over the chromosomes [18]. The density of the filtered gene set gene calls is displayed as green bars to the right of the chromosomes, with bar length indicating the number of genes per 400 kbp.

The display of the maize genome (Figure 2) illustrates the use of CViT for showing gene density, based on gene models generated as part of the *Zea mays* inbred B73 genome sequencing project [17]. The MaizeGDB use of CViT also includes a BLAST utility that displays color-coded hits on the reference genome with links to GBrowse for closer investigation.
